# Endovascular embolization for treating hemothorax: caveats and considerations

**DOI:** 10.1007/s12055-026-02202-y

**Published:** 2026-03-12

**Authors:** Indy Kelderman, Bernd Popko Teunissen, Chris Dickhoff, Martijn Ruben Meijerink, Wietse Pieter Zuidema

**Affiliations:** 1 Department of Trauma Surgery, Amsterdam University Medical Center, Meibergdreef 9, Room J1A-226-2, Amsterdam, 1105 AZ The Netherlands; 2 Department of Radiology and Nuclear Medicine, Amsterdam University Medical Center, Amsterdam, The Netherlands; 3 Department of Thoracic Surgery, Amsterdam University Medical Center, Amsterdam, The Netherlands

**Keywords:** Thoracic trauma, Hemothorax, Embolization, Endovascular, Coiling

## Abstract

Thoracic trauma is the third leading cause of death in all age groups. Around a quarter of trauma mortalities is related to thoracic trauma, with a portion of the patients developing a hemothorax requiring surgical intervention. Recently, endovascular embolization has seen a rise in usage as a less invasive alternative to surgical intervention in treating hemothorax. However, larger series and trials comparing efficacy and complication have yet to be completed. In this case series and narrative review of available literature, we described our experience with the endovascular treatment of hemothoraces, and performed a literature review of smaller series on efficacy and re-intervention rates of endovascular embolization compared to surgical intervention. Smaller described cohorts show efficacy rates of up to 93% of endovascular embolization in achieving hemostasis in traumatic hemothorax, with up to 68% needing surgical re-intervention. Based on our experiences and available literature, we propose a clinical algorithm when considering embolization instead of immediate surgical intervention.

**Supplementary Information:**

The online version contains supplementary material available at 10.1007/s12055-026-02202-y.

## Introduction

Thoracic trauma accounts directly for 10 to 30% of trauma-related deaths and is present in 60% of polytrauma patients [1]. In up to half of chest trauma patients, hemothorax occurs, with rib fractures increasing the odds sharply. Hemothorax can be life-threatening if left untreated due to hypovolemia and compromised venous return secondary to increased intrathoracic pressure. Next to immediate morbidity and mortality, hemothorax may also result in a longer hospital length of stay and later complications including empyema and fibrothorax. Rapid clinical assessment and personalized treatment are essential in lowering the overall morbidity, mortality, and hospital length of stay in this patient group.

Diagnosis and evaluation of a hemothorax rely on history, physical exam, and imaging, such as chest radiography and computed tomography (CT). CT may also be used in combination with angiography (CT-A). Chosen management of hemothorax depends on multiple factors, including but not limited to the size and type of hemothorax, the patient’s clinical condition, vital signs, institutional facilities, and experience. Initial management mainly consists of thoracic drainage. Massive or retained hemothorax are two other manifestations of hemothorax that need more aggressive management and are associated with higher mortality. Many proposed treatment options have been described in literature for treating these entities, but thoracotomy and video-assisted thoracic surgery (VATS) remain the preferred treatment options [2, 3]. Due to normal general anesthesia for thoracotomy compared to the more time-consuming single-lung anesthesia in VATS, regular thoracotomy is more viable in patients with hemodynamic instability compared to VATS [2]. However, thoracotomy is generally associated with more postoperative pain, higher risk of wound infections, and longer length of stay compared to VATS. Additionally, quick and minimally invasive transcatheter arterial embolization (TAE) has been found to be an alternative method in achieving hemostasis in traumatic hemothoraces.

With two cases from our institution’s experience describing the technical feasibility with later complications, and review of available literature we performed, we describe some matters to consider before performing endovascular embolization. We also propose an algorithm to support clinical decision-making when treating traumatic hemothoraces. After our experience with the procedure and the complications that followed, we hypothesized that despite achieving hemostasis, TAE does not allow for surgical exploration of the thorax, which may result in higher secondary intervention rates. A scoping literature review was conducted using the PubMed database. The aim was to map available evidence and identify key findings and knowledge gaps rather than to perform a systematic review. All articles in which at least five or more patients were treated with thoracic TAE were included. Hemothorax originating from non-thoracic wall injuries fall out of the scope of this algorithm.

## Case 1

Our first patient was a 71-year-old man who presented at our emergency department with dyspnea and pain on the right hemithorax after a fall from his bicycle, hitting a pole. The patient used dual antiplatelet therapy due to a recent coronary intervention. Due to suspected hemorrhagic shock, fluid resuscitation was started. Imaging showed a hemothorax on the right side; a thoracic drain was inserted, producing 2000 cc of blood. Despite fluid resuscitation, the patient remained tachycardic and hypotensive. The patient underwent CT with contrast, which showed a persistent hemothorax despite thoracic drainage, absence of rib fractures, and an arterial blush from the ninth intercostal artery on the right side. The patient was transferred to the hybrid operating room for endovascular embolization. During the procedure, a 5-French sheath was retrogradely inserted in the right femoral artery after local anesthetics. Angiography showed an active blush. A total of three coils were placed in the ninth right intercostal artery to achieve hemostasis as seen in Video 1. The procedure was performed without any complications, and the right femoral artery was closed with Angioseal^®^.

Following the procedure, vital signs improved and stabilized. During admission, drain production decreased vastly, and chest radiography showed mild atelectasis, improved air density, and partially well-defined diaphragm contours on the right hemithorax. The thoracic drain was removed, and after a brief period of observation, the patient was discharged in good clinical condition with return precautions. A few days after discharge, the patient presented to our emergency department with general malaise and dyspnea. Infection parameters were elevated, and CT confirmed empyema. Antibiotics were started, and the patient underwent washout and lung decortication by VATS. The procedure was performed without further complications, and the patient was discharged after 2 days.

## Case 2

Our second patient was a 30-year-old man who presented at our emergency department with tachypnea and low blood pressure after a stab wound in the left hemithorax. The patient had no prior medical history. Chest radiography showed a left-sided hemopneumothorax, and a thoracic drain was inserted, which produced 500 cc in the first hour. The patient underwent CT with contrast after drain placement, and a complete transection of the sixth left intercostal artery with active blush was seen. Serial measurements revealed a decrease of hemoglobin concentrations from 9 to 6 g/dL during observation in the emergency department. The patient was transferred to our hybrid operating room for endovascular embolization. Using the same technique as applied in the first case, angiography was used to localize the transection. Three coils were placed, and control angiography showed no blush after embolization. The procedure was performed without any complications. After the procedure, vital signs improved and remained stable. Drain production stopped, chest radiography showed fully expanded lung tissue without residual pneumo- or hemothorax, and serum hemoglobin concentrations remained stable. The drain was set to waterseal for 24 h and was removed after control radiography showed no residual pneumo- or hemothorax, with a well-defined diaphragm bilaterally. The patient was discharged in good clinical conditions with return precautions.

A few days after discharge, the patient presented to the emergency department again with shortness of breath. Supplemental oxygen was started. Chest radiography showed pleural fluid and a small pneumothorax. A chest drain was placed, which produced 200 cc of serous fluid immediately, and the patient was readmitted. A few days after admission, the drain was successfully set to waterseal for 24 h again, after which the drain was removed. The patient was discharged in good clinical condition.

## Discussion

In this small case series, we describe our institution’s experience with TAE in treating traumatic hemothorax. In our first patient, dyspnea was reported and hemothorax without evident rib fractures was confirmed. Thoracic drainage was started, and as massive hemothorax was present, adjuvant treatment was indicated. As CT with contrast showed an isolated arterial blush, embolization with coils was chosen and fast hemorrhage control was successfully achieved. During admission, the patient showed improved clinical condition, low drain production, and largely improved chest radiography. The patient was subsequently discharged. However, the patient was later readmitted and needed secondary surgical washout through VATS.

This clinical course aligns with findings from the available literature. In several retrospective series using various embolization techniques, primary hemostasis was achieved in the majority of cases, with reported technical success rates ranging from 80 to 100%. However, rates of secondary surgical intervention varied widely between 7.4 and 68% [3–8]. Table 1 summarizes the characteristics and outcomes of the included cohorts. These studies differ considerably in terms of etiology, bleeding source, outcome definitions, and reporting of secondary surgical interventions.

**Table 1:** A summary of cohorts found in literature using TAE in at least 5 patients with traumatic hemothorax

**Name**	**Year published**	**Study design**	**Number of patients**	**Setting/population**	**Method of embolization**	**Primary efficacy rates**	**Surgical re-intervention rates**
**Chemelli **[3]	2009	Retrospective cohort	24 patients (11 traumatic, 13 iatrogenic)	Traumatic or iatrogenic hemothoraces	Coiling or embolization	87.5% (90.9% traumatic, 84.6% iatrogenic)	Not mentioned
**Hagiwara **[4]	2008	Retrospective cohort	5 patients	Traumatic hemothoraces	Gelfoam	100%	Not mentioned
**Lee **[5]	2021	Retrospective cohort	76 patients (68 survived > 48 h after TAE, this group was analyzed)	Traumatic hemothoraces	Based on radiologist’s preference	92.6%	7.4% required thoracotomy for hemostasis, later re-interventions not mentioned
**Stampfl **[6]	2014	Retrospective cohort	19 patients (13 hemothorax, 6 extrahepatic)	All traumatic, iatrogenic, and spontaneous hemorrhage from intercostal arteries	Based on radiologist’s preference	80%	68.4%
**Tamburini **[7]	2019	Retrospective cohort	30 patients	All traumatic, iatrogenic, and spontaneous hemothorax	Based on radiologist’s preference	87%	43%
**Whigham **[8]	2002	Retrospective cohort	18 patients (12 embolization, 2 surgical ligation, 4 observation)	Traumatic hemothorax from internal mammary artery	Coils or gelfoam	91.6%	8% required thoracotomy for hemostasis, later re-interventions not mentioned

**Fig. 1:**
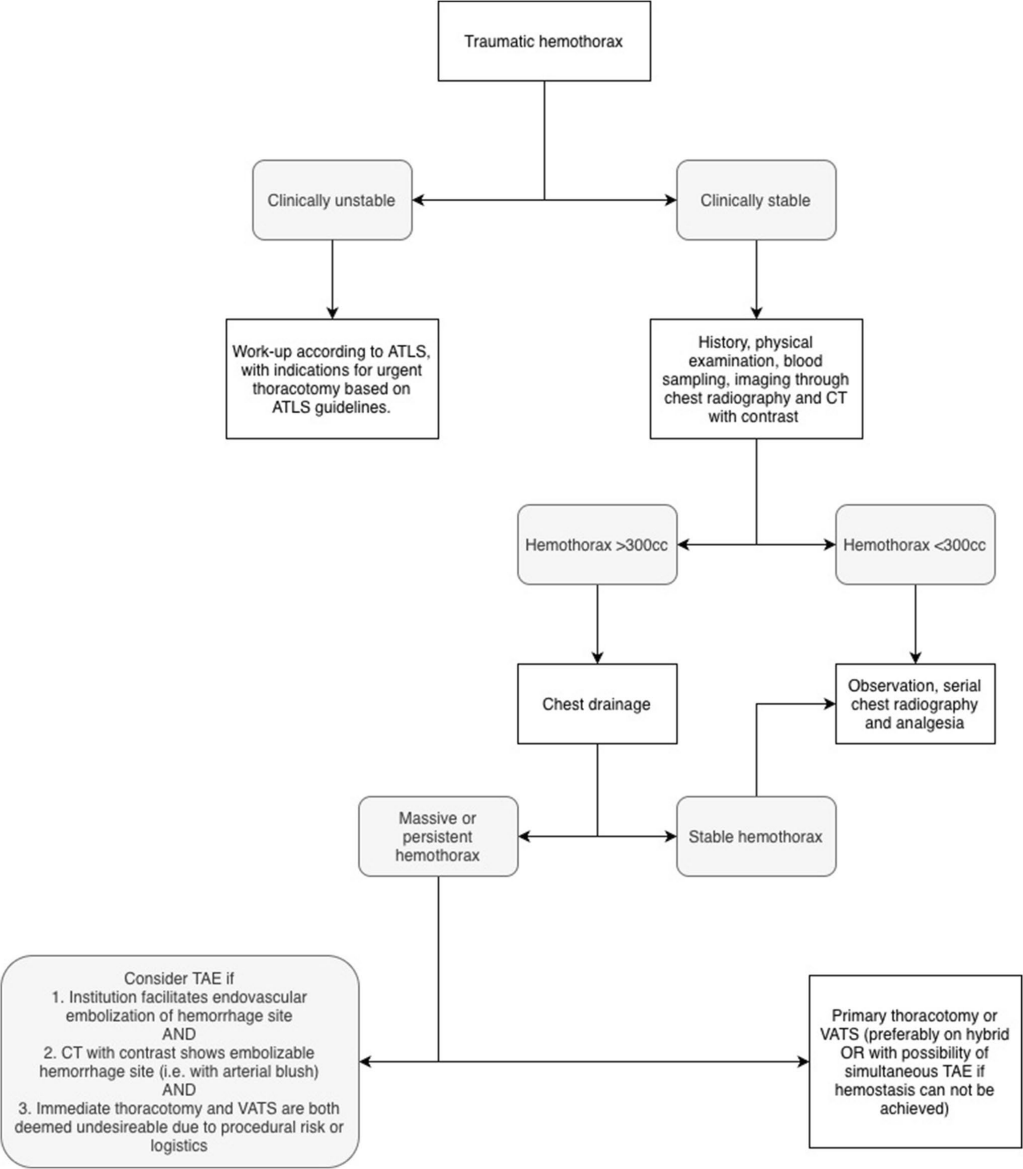
A proposed algorithm in defining the role of using TAE in treating traumatic hemothorax, based on our interpretation of currently available literature

In the cohort reported by Stampfl et al. 13 out of 19 (68.4%) patients required surgical intervention after embolization, either due to hematoma evacuation or technical failure [6]. Other series by Chemelli, Hagiwara, Lee, and Whigham mention complications such as technical failure, but do not specify occurrence of retained hemothorax or empyema requiring later surgical intervention [3–5, 8]. Given the heterogeneity of cohorts, small sample sizes, and methodological differences, only limited conclusions can be drawn beyond initial technical feasibility or efficacy. Literature shows that early VATS in retained hemothorax reduces hospital length of stay, intensive care unit days, and the percentage of pleural infections [9]. Although TAE achieves high initial success, the need for surgical re-intervention in a considerable number of patients suggests that an early surgical approach may be preferable in selected cases. A proposed algorithm for the initial management of hemothorax requiring adjuvant interventions is shown in Fig. 1.

Our second case involved a patient who was readmitted several days after discharge. During the initial admission, chest radiography had shown adequate lung expansion and minimal drain output. This suggests that the pleural collection identified at readmission developed in the days following discharge, after drain removal. The most likely explanation included incomplete evacuation with loculated residual pleural fluid or a small rebleed from the initial admission.

Large reviews of readmission rates after thoracic trauma have been performed in the past. In a nation-wide study from the United States, Kirchberg et al. found that 794 (5.7%) out of 13,903 patients admitted for traumatic hemothorax in 2017 had to be readmitted within 30 days due to chest-related pathologies [10]. A total of 1574 patients initially received thoracic drainage, with 287 (18%) requiring invasive re-intervention (i.e., thoracic drainage, thoracotomy, or VATS). As embolization was not described as intervention, percentage of these patients requiring readmission is not described in this study.

While different smaller studies describe high initial technical success rates, surgical re-intervention rates after TAE remain poorly described. When considering TAE for achieving hemostasis, patient characteristics, comorbidities, and individual and institutional experience should be taken into consideration. Immediate, aggressive surgical thoracotomy or VATS may be preferred over TAE for combining achieving hemostasis and drainage, with TAE acting as a supporting procedure during thoracotomy or VATS. Lastly, larger trials exploring incidence rates, efficacy rates of both treatment modalities, and optimal treatment strategies in traumatic hemothorax are needed to improve our knowledge and improve outcomes in these patients.

## Conclusion

While TAE is safe for achieving initial hemostasis in traumatic hemothorax, immediate aggressive surgical intervention may be preferable to reduce delayed complications, the likelihood of secondary procedures, and prolonged hospital stay.

## Supplementary Information

Below is the link to the electronic supplementary material.

ESM 1 (MOV 1.98 MB)

## Data Availability

The authors confirm that the data supporting the findings of this study are available within the article, with clinical data being available on request.
